# Induction-free recombineering for simple targeted gene-deletions in various mycobacteria

**DOI:** 10.1128/spectrum.01897-25

**Published:** 2025-08-04

**Authors:** Yahav Bracha, Daniel Barkan

**Affiliations:** 1 The Koret School of Veterinary Medicine, Robert H. Smith Faculty for Agriculture, Food and Environment, The Hebrew University of Jerusalem 108750, Rehovot, Israel; CNRS-University of Toulouse, Toulouse, France

**Keywords:** *Mycobacterium tuberculosis*, gene deletion, technique, *Mycobacterium marinum*

## Abstract

Gene deletion is a valuable tool for phenotypic characterization in bacteriology, but in mycobacteria, generating deletion mutants remains a cumbersome and time-consuming process. Here, we present a modification to the widely used recombineering method in mycobacteria. By expressing the recombineering-promoting proteins from a single-copy episomal plasmid, we could use a constitutive promotor. This approach eliminates the need for induction prior to electroporation of the targeting substrate (linear DNA fragment comprised of homologous regions to the gene of interest, flanking a selection marker), and shortens the subsequent plasmid curing process. We successfully demonstrated this method in *Mycobacterium tuberculosis*, *M. marinum*, *and M. abscessus*.

**IMPORTANCE:**

Although several techniques exist for generating gene-deletion mutants in mycobacteria, these procedures remain limited to laboratories more specialized in molecular biology. Here we present a very simplified procedure, which is a modification on a well-tried technique. Our proposed procedure makes genetic manipulation in mycobacteria more accessible to a greater number of researchers throughout the world, including those with less advanced molecular biology expertizse.

## INTRODUCTION

Targeted gene deletion is an invaluable technique in bacterial genetic research, yet in all mycobacteria, and especially the *Mycobacterium tuberculosis* (Mtb), *Mycobacterium marinum,* and *Mycobacterium abscessus*, it remains a tricky procedure that is time consuming, and in many cases, frustrating. Several methods to produce deletion mutants have been published. However, all of them have drawbacks, including low yield (two-step allelic exchange with negative selection [1, 2]), or relatively complicated and lengthy procedures (temperature-sensitive mycobacteriophage-based techniques [3], and, to an extent, the ORBIT system [4]). The most widely used method is based on the recombineering system published in 2007 (5). In this method, firstly, a linear DNA fragment consisting of a resistance marker of choice flanked on both sides by ~500–1,000 nt-long flanking regions of the targeted gene is prepared, either by consecutive PCRs or by cloning. This fragment (“targeting substrate,” or “allelic exchange substrate,” AES) ranges in length between 1,500 and 3,500 base pairs depending on the selection marker and the length of the flanking regions. Secondly, an episomal plasmid with recombination-promoting genes is inserted by electroporation into the target mycobacterium (*M. smegmatis, M. abscessus, M. marinum, or M. tuberculosis*), and selected by another marker (usually kanamycin). These recombination-promoting enzymes (genes *gp60, gp 61*), originating from mycobacteriophages, are expressed from an acetamide-responsive promoter as their constitutive expression in high levels is toxic to the bacterium. In the original paper from 2007, this plasmid was named pJV53. Prior to electroporation of the “targeting substrate” into the target bacteria, the recombineering genes must be induced for one generation time. This necessitates not only the addition of acetamide, but also the removal of the oADC (as dextrose inhibits the acetamide promoter) and substituting it by succinate. The induced bacteria are then electroporated with the AES and plated on the appropriate selection marker. The resulting colonies (if any) are examined for correct recombination (versus illegitimate recombination or spontaneous background) by appropriate PCR. The correct deletion mutant is then cured of the pJV53 plasmid by serial passage and counter-patching.

This method was (and still is) widely used to create targeted deletion mutants in various mycobacteria, including *M. abscessus, M. marinum,* and Mtb. However, two factors complicate its use: The first is the need for acetamide induction. We noticed the addition of acetamide and the subsequent induction of *gp60-61* for one generation time tends to reduce the viability of the bacteria. Additionally, not only is the media with oADC need to be removed, but the original protocol also calls for substituting it by succinate-based media, which supports growth to a much lesser extent. These factors complicate the pre-electroporation induction. Secondly, after a successful deletion is performed, the pJV53 plasmid needs to be lost. This is sometimes a lengthy procedure. For Mtb, that retains plasmids very well and is also a slow grower, the process of curing from pJV53 may be long and time consuming.

Here, we present a simple tweak to this method, allowing the elimination of the acetamide-induction step (and thus, also the media change from oADC to succinate-based), and greatly simplifying the plasmid loss procedure. We successfully obtained targeted deletion mutants across multiple mycobacteria, with simple and quick plasmid cure.

### Overview of proposed tweak

**Fig 1:**
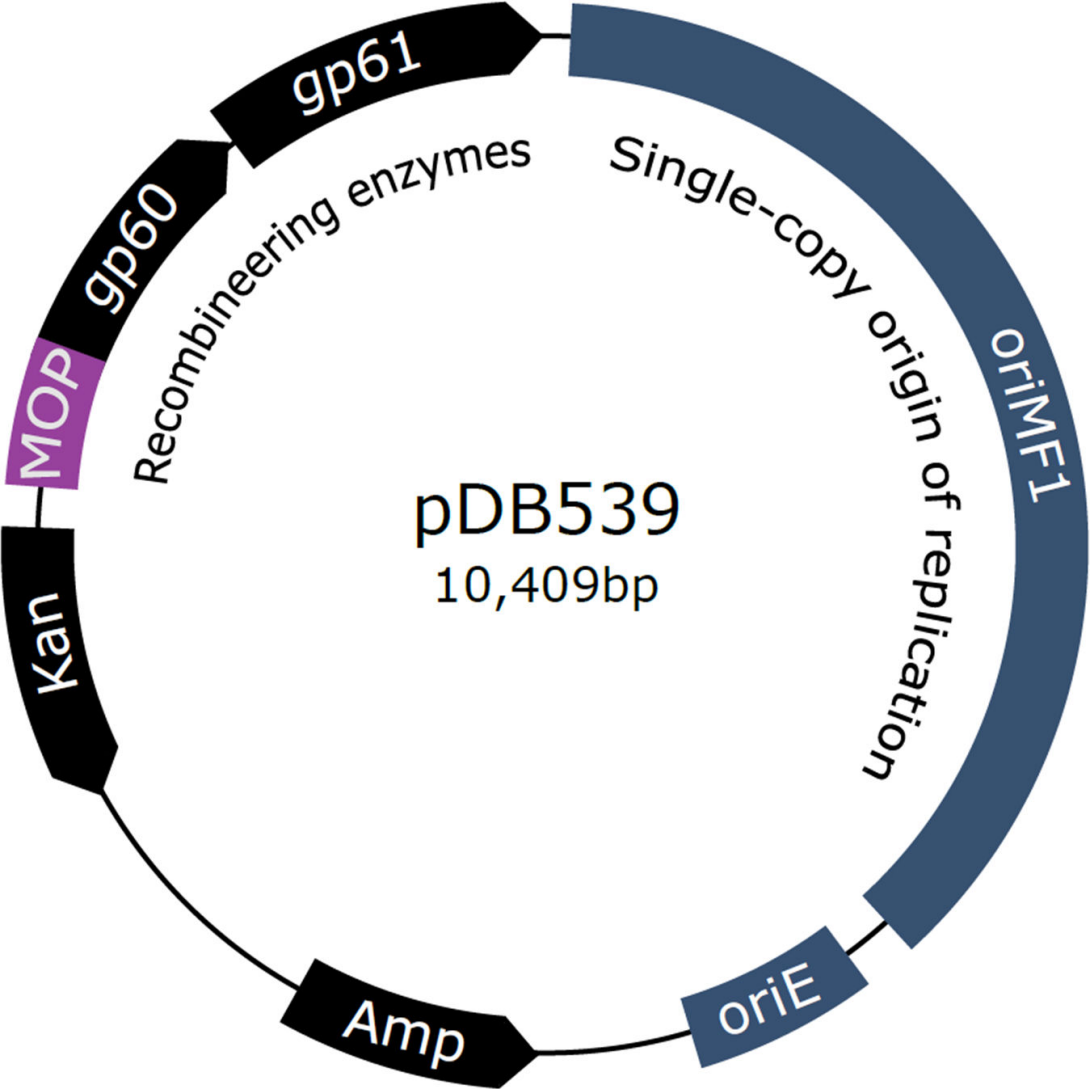
A diagram of the constructed pDB539 plasmid. The recombineering genes (*gp60, gp61*, also known as *che9c, che9c2*) are expressed from the MOP constitutive promoter. The mycobacterial origin confers single-copy plasmid number.

We PCR-amplified the *gp60* and *gp61* genes from the original pJV53 plasmid, and placed them under the constitutive MOP promotor. This construct was cloned into an *E. coli*/mycobacteria shuttle vector with the MF1 mycobacterial origin (not the *oriM* used in pAL5000-based shuttle vectors). The resulting plasmid was named pDB539 (Fig. 1). The MF1 origin, isolated from *M. fortuitum* in 1999 (6), is unique in the sense it is a single copy plasmid, and is rapidly lost from mycobacteria (including Mtb) when no active selection pressure is applied (7). Placing *gp60-61* on this single (rather than multi-) copy plasmid enabled the expression from a constitutive promoter, thus abolishing the need for induction. At the same time, loss of the plasmid after a successful deletion procedure is rapid—the loss process begins immediately after electroporation (when the bacteria are plated on the deletion selection marker), so when colonies appear, the vast majority of the bacteria have already lost it. Otherwise, the procedure is identical to the previously described one, with similar generation of a linear DNA AES (“targeting substrate”) and electroporation.

## RESULTS

### Deletion of the small RNA B11 from *M. abscessus*

**Fig 2:**
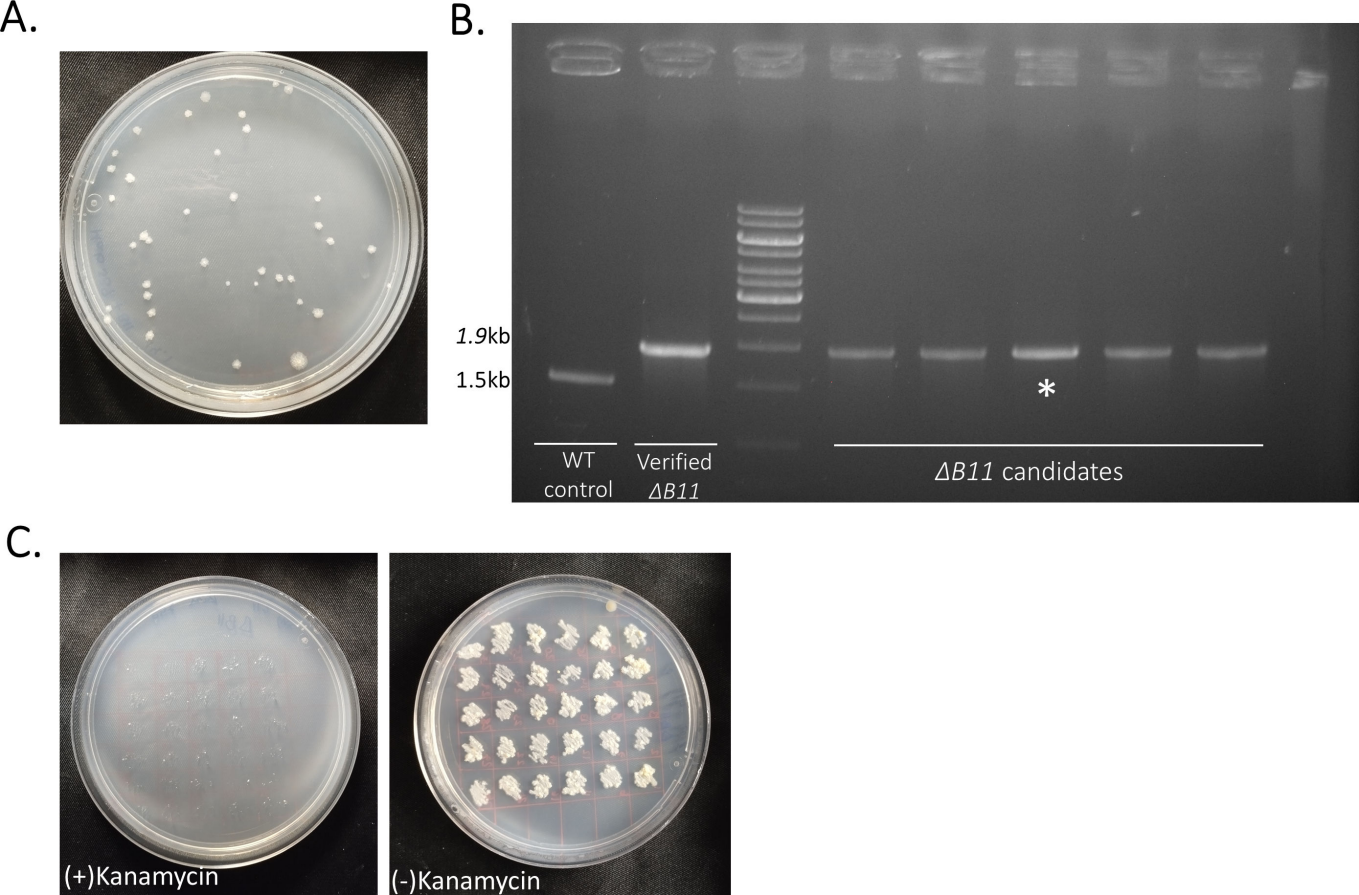
Generation of multiple ΔB11 mutants in *M. abscessus*. (**A**) the colonies obtained after AES electroporation into *M. abscessus*^pDB539^ and plating on zeocin 66 µg/mL. all 39 colonies on the plate have the rough morphology characteristic of the B11 deletion. (**B**) Five random colonies from the plate in (**A**) were tested by PCR, with primers yielding a 1.5 kb fragment in wt, and a 1.9 kb fragment in a correct deletion mutant. A verified ΔB11 mutant was used as a positive control (37603560). The colony marked with (*) was chosen to test pDB539 loss. (**C**) the * colony was plated for singles, and 30 of them were patched on plates with (right) and without (left) kanamycin. None retained kanamycin resistance, indicating pDB539 was successfully lost.

To test the feasibility of our proposed tweak, we started by deleting a gene we knew could be deleted from *M. abscessus*. We previously deleted the sRNA B11 from *M. abscessus* (using phage-based transduction) (8). Putative identification of correct deletion mutants on agar plates is facilitated in this case by a very distinct “rough” colony morphology of the mutants. Here, we introduced pDB539 into WT *M. abscessus* and selected correct transformants (on kanamycin 240 µg/mL). The presence of the plasmid was verified by targeted PCR of the *kanamycin^R^* and the *gp60-61* genes. In parallel, a linear “targeting substrate” targeting the B11 sRNA (flanking regions of 1,700 and 1,800 bp, zeocin resistance) was prepared by PCR. *M. abscessus*^pDB539^ was electroporated by this linear construct and plated on agar plates with zeocin 66 µg/mL. After 6 days, a total of 37 colonies appeared on the plate, all with the characteristic rough morphology (Fig. 2A), suggesting that they were all correct deletion mutants. Of these 37 colonies, five were also examined by PCR, and were confirmed to be correct (Fig. 2B). Immediately following confirmation, one of the mutants was plated for single colonies, and 30 of them were examined for loss of pDB539. Patching on plates with and without kanamycin showed all 30 examined clones have successfully lost the pDB539 plasmid (Fig. 2C).

### Deletion of *mmar_3011-4* from *M. marinum^moffett^*

**Fig 3:**
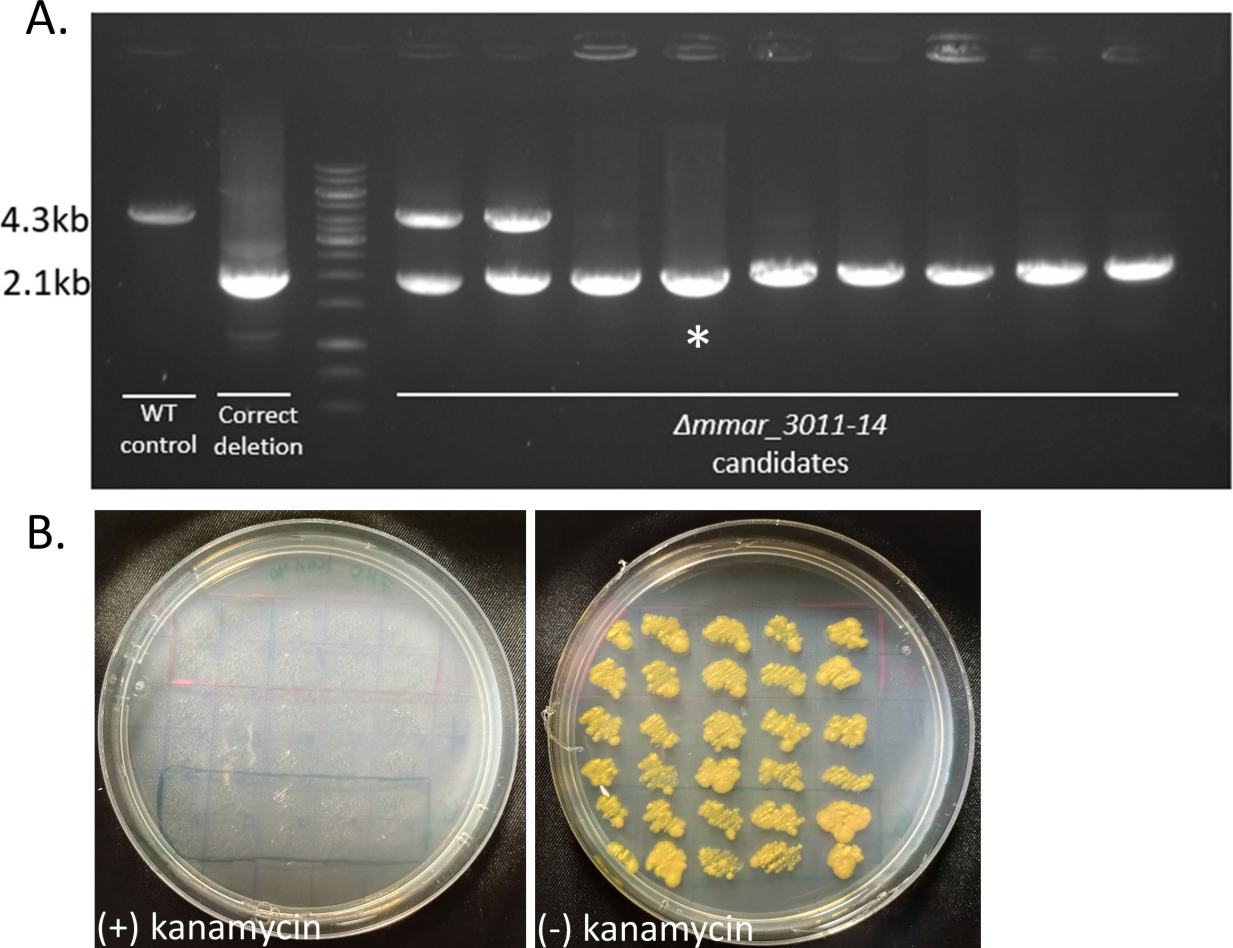
Deletion of *mmar_3011-14* from *M. marinum*. (**A**) Nine candidate colonies that grew on hygromycin 50 µg/mL were tested by PCR that yields a 4.3 kb fragment in wt, and a 2.1 kb fragment in a correct deletion mutant. WT and the AES itself were used as controls. The colony marked with (*) was randomly chosen for continuation. (**B**) The (*) colony was immediately plated for singles, and 30 resulting single colonies were patched with (left) and without (right) kanamycin.

Targeted gene deletion in *M. marinum* is more challenging than in most other mycobacteria, probably due to the high frequency of illegitimate recombination. We previously deleted the *leuD* gene from *M. marinum* using specialized phage transduction, and encountered high rates of background colonies. Here, we opted to delete the sugar transporter encoded by *mmar_3011-4*. We first introduced pDB539 into WT *M. marinum^moffett^*. We prepared a hygromycin-based deletion construct with flanking regions of 1,000 and 900 bp, and performed the electroporation procedure as described before. We obtained several dozens of colonies on the hygromycin 50 µg/mL plates, of which we picked and sub-cultured nine, and tested them by PCR (Fig. 3A). As seen, 7/9 colonies were confirmed to be correct deletion mutants, whereas another two were probably illegitimate recombination events, yielding both the product expected from WT and the one expected from deletion mutants. Immediately after PCR confirmation, one of the correct clones was plated for single colonies directly from the subculture tube. Thirty single colonies were then tested for kanamycin sensitivity (i.e., –pDB539 loss), and all of them (30/30) were found to have lost the plasmid (Fig. 3B).

In a separate experiment, we opted to delete the transcription factor *mmar_1132* (*whiB3*) from *M. marinum*, using a similar strategy. After electroporation and plating on hygromycin plates, three colonies appeared. All three were tested by PCR to confirm the deletion, and all were found to be correct deletion mutants.

### Deletion of *rv0275* from *M. tuberculosis H37Ra*

The drawbacks of the “classic” pJV53-based method as originally published are most pronounced in Mtb. As Mtb must be grown in oADC, the media have to be thoroughly replaced before the induction with media completely devoid of oADC, and supplemented by succinate, which supports Mtb growth to a lesser extent. Induction by acetamide for one generation (24 hours) is detrimental to the bacteria, and induction time needs to be adjusted. Since Mtb retains plasmids very efficiently even without selection, pJV53 loss after completion of the deletion can be time consuming and difficult, especially if the deletion mutant also has a slow-growth phenotype. We previously attempted to delete *rv0275c* (a non-essential TetR-like transcription factor) from Mtb *H37Ra* using pJV53, but only obtained illegitimate recombination mutants.

**Fig 4:**
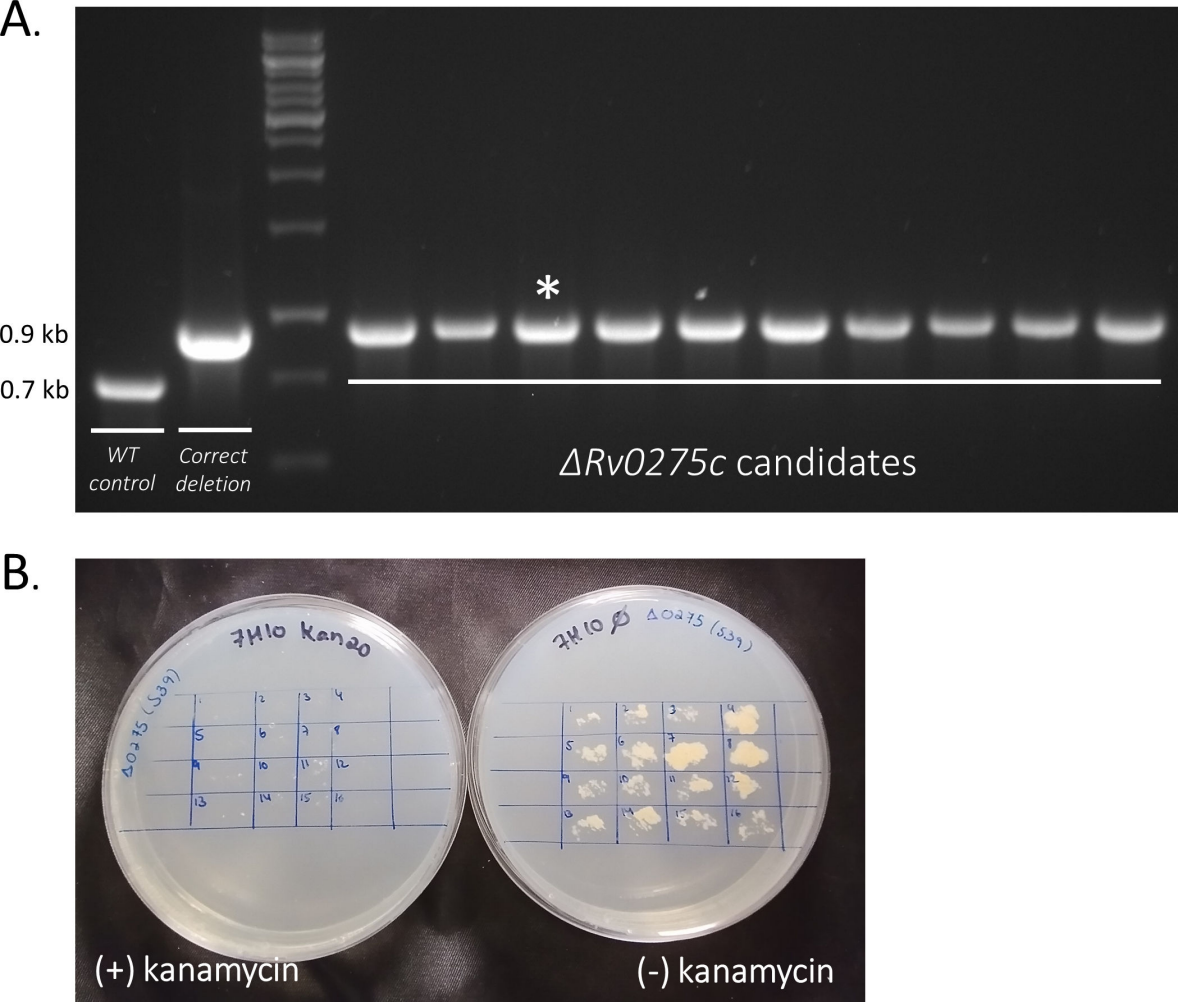
Deletion of *rv0275* from Mtb H37Ra. (**A**) A PCR on 10 random candidate colonies was performed, yielding 0.7 kb fragments in WT genotype, and 0.9 kb in a correct deletion mutant. WT and the AES itself were used as controls. The (*) colony was chosen for continuation. (**B**) the (*) colony was plated for singles, and 16 of them were patched with (right) and without (right) kanamycin. None retained kanamycin resistance.

We now introduced pDB539 into Mtb by kanamycin (20 µg/mL) selection, and verified the transformants by PCR for *kanamycin^R^* and for the *gp60-61* genes. We then used a zeocin-based “targeting substrate”, with flanks spanning 720 and 700 bp to target *rv0275c*. No media change and no induction were used. After electrotransformation, bacteria were plated on zeocin 25 µg/mL. Dozens of colonies appeared after 24 days, out of which 10 were subcultured for analysis. Once grown, the 10 isolates were tested by PCR, and all (10/10) were found to be correct deletion mutants (Fig. 4A). As before, one of the confirmed deletion mutants was immediately plated for single colonies, and 16 colonies were tested for plasmid loss. Subsequently, 16/16 were found to have lost the pDB539 plasmid (Fig. 4B).

Applying a similar procedure, we were also able to delete *rv2686-88c,* a putative ciprofloxacin efflux pump, with 6/6 candidates tested found to be correct. This deletion was done using hygromycin 50 µg/mL for selection.

## DISCUSSION

### Selection marker choice

In *M. abscessus*, we found zeocin 50–66 µg/mL to provide stringent selection. Apramycin is also reported to work well in *M. abscessus*. Hygromycin necessitates very high concentrations (up to 1,000–2,000 µg/ml), and even then works relatively poorly. In *M. marinum,* we used hygromycin 50 µg/mL with very good selection and minimal background. However, when we tried zeocin 50 µg/mL, we got substantial background. We do not know if this is a general feature, or a specific problem with our lab strain. We did not try apramycin. For Mtb, we used zeocin 25 µg/mL for one deletion, and hygromycin 50 µg/mL in the other. We did not attempt apramycin.

### Electroporation procedure

As double cross-over recombination (leading to deletion creation) are a rare events, a very efficient electroporation procedure is essential. This is also true for classic pJV53-based procedures. We therefore recommend positive (a mycobacteria-competent plasmid with the same selection marker) and negative control (a plasmid with a different selection marker) plates. If the positive control plate does not show a very high efficiency electroporation, chances for a successful recombination in the “experiment” plate are lowered. Based on our prior observation that mycobacteria with defective cyclopropanation of mycolic acids have much higher transformation efficacy (9), and that dioctylamine can inhibit cyclopropanation (and hence transformation efficacy) (9), we opted to add dioctylamine (20–40 µM) to the growth media of all mycobacteria 1–2 days prior to the electroporation. Although we did not formally test whether this increased transformation efficacy, we believe (by the positive control plates) there was a substantial increase, which may have contributed to the success ratio of the deletion. Electroporations were otherwise performed as widely described, in cold conditions for *M. abscessus*, and room temperature conditions for *M. marinum* and Mtb.

### Overall success

As all techniques for gene deletion in mycobacteria, this system is not 100% proof. We made two attempts to delete *rv2038-41* from Mtb, but neither were successful. For *rv0275*, two attempts were made—the first yielded no colonies at all, whereas the seconds yielded dozens of colonies, of which only 10 were tested and all were correct. Regarding *rv2686-88,* we originally attempted to delete it using pJV53, but only obtained illegitimate recombination or background. When applying this new method, we obtained multiple colonies, out of which six were examined and found correct. For the deletion of *mmar_1132* from *M. marinum*, we conducted two attempts—one yielded no correct mutants, whereas the second yielded three. For *mmar_3011-4*, the first and only attempt yielded dozens of colonies, of which the majority (7/9 examined) were correct. This suggests that there is substantial variation in the success rate between attempts, even with exactly the same AES construct. We hypothesize that this is mostly related to transformation efficiency of each individual attempt.

Overall, we believe this technique, a tweak of an existing and successful system, can substantially simplify the procedure of targeted gene deletions in mycobacteria and make it more accessible and “less intimidating” for a larger number of research labs throughout the world—including those that do not specialize in molecular biology. We see it as an important addition to the genetic tool-box in mycobacteria research—not aimed at complete replacement of existing techniques, but a valuable additional option. Obviously, we offer the pDB539 to any researcher who requests it.

## MATERIALS AND METHODS

Most of the methods and procedures of this work were described in detail in the relevant section. Some general procedures are described here.

### Bacteria

*M. abscessus* was grown in 7H9/glycerol/tween, as previously widely described. *M. marinum* and Mtb H37Ra were grown in 7H9/glycerol/tween/ADS, as previously widely described. Solid agar plates were prepared by omitting the Tween, and adding 15 g/L of agar.

### Antibiotics

For *E. coli*, we used zeocin 33 µg/mL, hygromycin 150 µg/mL, and kanamycin 40 µg/mL. For M. abscessus, we used zeocin 66 µg/mL, and kanamycin 240 µg/mL. For M. marinum, we used kanamycin 40 µg/mL, and hygromycin 50 µg/mL. For Mtb, we used zeocin 25 µg/mL, hygromycin 50 µg/mL, and kanamycin 20 µg/mL.

### Electroporation

Mycobacteria were grown in 7H9-based media, at a volume of 50 mL, to an O.D_600_ of 0.5–0.6. in some of the experiments, dioctylamine 20 µM was added when the O.D_600_ was *sirca* 0.1. Before electroporation, bacteria were washed twice in glycerol 10% (cold for *M. abscessus*, room temperature for *M. marinum* and Mtb), and electroporated as widely described. Bacteria were plated on the appropriate antibiotic after recovery of one generation time.
